# Molecular Mechanism of Hippo–YAP1/TAZ Pathway in Heart Development, Disease, and Regeneration

**DOI:** 10.3389/fphys.2020.00389

**Published:** 2020-04-23

**Authors:** Xiaoqing Chen, Yilang Li, Jiandong Luo, Ning Hou

**Affiliations:** ^1^Key Laboratory of Molecular Target & Clinical Pharmacology, School of Pharmaceutical Sciences and the Fifth Affiliated Hospital, Guangzhou Medical University, Guangzhou, China; ^2^Guangzhou Institute of Cardiovascular Disease, Guangzhou Key Laboratory of Cardiovascular Disease, and The Second Affiliated Hospital of Guangzhou Medical University, Guangzhou, China

**Keywords:** YAP1/TAZ, Hippo pathway, post-translational modification, heart development, cardiac disease, regeneration

## Abstract

The Hippo–YAP1/TAZ pathway is a highly conserved central mechanism that controls organ size through the regulation of cell proliferation and other physical attributes of cells. The transcriptional factors Yes-associated protein 1 (YAP1) and PDZ-binding motif (TAZ) act as downstream effectors of the Hippo pathway, and their subcellular location and transcriptional activities are affected by multiple post-translational modifications (PTMs). Studies have conclusively demonstrated a pivotal role of the Hippo–YAP1/TAZ pathway in cardiac development, disease, and regeneration. Targeted therapeutics for the YAP1/TAZ could be an effective treatment option for cardiac regeneration and disease. This review article provides an overview of the Hippo–YAP1/TAZ pathway and the increasing impact of PTMs in fine-tuning YAP1/TAZ activation; in addition, we discuss the potential contributions of the Hippo–YAP1/TAZ pathway in cardiac development, disease, and regeneration.

## Introduction

The Hippo pathway, originally identified in the *Drosophila* genus, is a highly conserved kinase cascade that regulates organ size (Harvey et al., 2003; Pan, 2007; Hayashi et al., 2015). The transcriptional coactivator Yes-associated protein 1 (YAP1; homolog of Yorkie) was first identified as a binding partner of the SH3 domain of c-yes; the YAP1 and its paralog PDZ-binding motif (TAZ; also known as WW–domain-containing transcription regulator 1 [WWTR1]), are both downstream effectors of Hippo signaling (Sudol, 1994). This pathway has been implicated in diverse biological functions, both in *Drosophila* and in mammals; these include cell proliferation, apoptosis, organ-size control, and cancer progression (Mo, 2017). Recent reports have revealed the critical role of YAP1/TAZ in cardiac development, regeneration, and stress response; however, there are some inconsistent and even contradictory results that warrant further investigation (Zhou et al., 2015). Interestingly, recent studies have identified a variety of post-translational modifications (PTMs) to YAP1/TAZ, such as phosphorylation, O-GlcNacylation, methylation, and ubiquitination, which offers an opportunity to control the Hippo–YAP1/TAZ pathway. In this review, we summarize the overall picture of the Hippo–YAP1/TAZ pathway; in particular, we highlight the novel discoveries with regard to PTM-related regulation and the function of the YAP1/TAZ pathway in cardiac development, disease and regeneration.

## Overview of the Hippo–YAP1/TAZ Pathway

### The Canonical Hippo–YAP1/TAZ Pathway in Mammals

**FIGURE 1:**
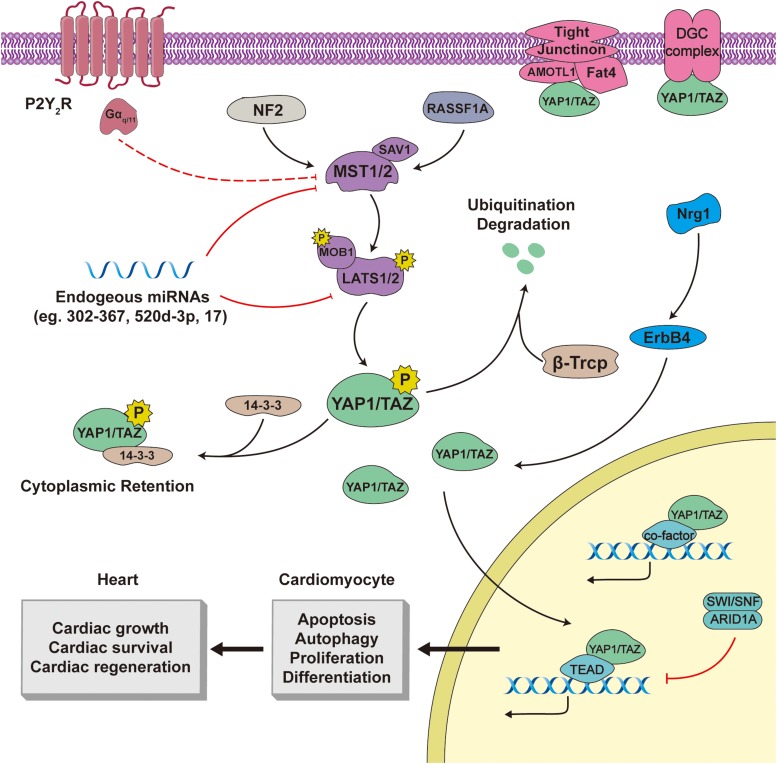
The Hippo–YAP1/TAZ pathway in cardiac biology. Both YAP1 and TAZ are phosphorylated by core components, including Mst1/2, SAV1, Lats1/2, and MOB1, of the canonical Hippo pathway, and are subsequently degraded or stranded in the cytoplasm. Active YAP1/TAZ translocate to the nucleus, bind to transcriptional partners, and modulate downstream output in cardiac tissue. In addition, the Hippo-YAP1/TAZ pathway is regulated by several upstream pathways and cell-cell junctions. Line arrows indicate activation, whereas connector lines imply inhibition.

The components of the Hippo pathway in mammals are highly consistent with those in *Drosophila*, including sterile 20-like protein kinases (MST1/2; homologs of D. Hpo), salvador family protein 1 (SAV1; which contains a WW domain), large tumor suppressors (LATS1/2; homologs of Wts), Mps one binder kinase activator-like 1A/1B (MOB1; orthologs of Mats), YAP1/TAZ, and other peripheral proteins (Figure 1).

The upstream signals and stressors of the Hippo pathway include cell polarity, energy stresses, G–protein-coupled receptors (GPCRs), and stiffness of the extracellular matrix (ECM) (Meng et al., 2016). On stimulation, MST1/2 and its adaptor protein SAV1 are phosphorylated leading to their activation; subsequently, these stimulate the phosphorylation, and activation of the LATS1/2–MOB1 complex. Through an interaction between the PPxY (PY) motifs of LATS1/2 and the WW domains of YAP1/TAZ, the activated LATS1/2 can induce phosphorylation of YAP1 (mouse YAP1 at Ser112, human YAP1 at Ser127) and TAZ (Ser89) (Zhao et al., 2007). After phosphorylation, YAP1/TAZ binds to the 14-3-3 protein, which induces cytoplasmic retention of YAP1/TAZ. Moreover, phosphorylated YAP1/TAZ is likely to undergo ubiquitination degradation that is dependent on the β-transducin repeat-containing E3 ubiquitin protein ligase complex (SCF^β–TRCP^) (Dong et al., 2007; Zhao et al., 2007).

When Hippo signaling is inactive, the YAP1/TAZ are activated and mainly localized in the nucleus, where they produce downstream biological effects. The YAP1/TAZ do not harbor DNA-binding domains; instead, these interact with transcriptional partner proteins to initiate or impede transcription. The transcriptional partners of YAP1/TAZ that have been identified thus far include transcriptional enhanced associate domain proteins (TEADs), members of the Smad family (Anorga et al., 2018; Ben Mimoun and Mauviel, 2018; Miskolczi et al., 2018; Qin et al., 2018), and p63/p73 (Levy et al., 2008; Kim et al., 2018). The onus of the YAP1/TAZ biological output rests with the transcriptional partners and the target genes that they modulate.

### Downstream Output of YAP1/TAZ

As orthologs of *Drosophila* Scalloped, the TEAD family of proteins in mammals comprise YAP1/TAZ- and DNA-binding domains, which facilitates the construction of a YAP1/TAZ–TEADs co-transcription complex (Li et al., 2010). Recent studies investigating YAP1/TAZ mechanisms have identified some target genes of the YAP1/TAZ–TEADs complex. The *glucose transporter 1* (*Glut1*) promotor contains TEAD-binding motifs that allow *Glut1* to be regulated by the YAP1–TEAD1 transcriptional complex, which enhances cell glycolysis in breast cancer cells (Valis et al., 2016; Lin and Xu, 2017). Moreover, GLUT1, a transmembranous protein, participates in cell glucose uptake (Wang et al., 2017). Left ventricular hypertrophy is characterized by enhanced GLUT1 expression and basal glucose uptake in heart tissues. Cardiac-specific overexpression of GLUT1 can retard the progression of heart failure and reduce mortality associated with pressure overload (PO) (Liao et al., 2002). Thus, YAP1/TAZ may have a cardioprotective effect by enhancing GLUT1 expression. In breast cancer cells, the *G2 and S phase-expressed 1* (*GTSE1*) acts as a novel target gene of the YAP1–TEAD4 complex, which forms cell protrusions and boosts cell migration (Stelitano et al., 2017). The GTSE1 is a microtubule-associated protein that is involved in cell proliferation, invasion, and migration (Liu et al., 2019). Therefore, through regulation of GTSE1, YAP1 facilitates epithelial-to-mesenchymal transition (EMT) in cancer cells.

Furthermore, the YAP1/TAZ–TEAD complex can recruit and bind to transcription enhancers and/or mediators to potentiate and blunt transcription. Prox1, a major regulator of lymphangiogenesis, is regulated by the YAP1/TAZ–TEAD complex (Harvey et al., 2005; Cho et al., 2019). The YAP1/TAZ–TEAD transcriptional complex recruits the nucleosome remodeling and histone deacetylase (NuRD) complex to negatively modulate *Prox1*; this was shown to reduce pathological lymphangiogenesis and maintain the lymphatic valve postnatally (Cho et al., 2019). In addition, the YAP1/TAZ, together with histone deacetylase 7 (HDAC7) and TEAD, binds to the *COX-2* promoter and attenuates *COX-2* transcription in order to inhibit IL-1β-induced cell migration and invasion (Zhang et al., 2018a). Based on its tyrosine phosphorylation status, parafibromin (a nuclear scaffold protein) selectively interacts with YAP1 or TAZ. Phosphorylated parafibromin binds to the YAP1-TEAD complex, whereas dephosphorylated parafibromin combines with the TAZ-TEAD transcriptional activator (Tang et al., 2018). YAP1 and TAZ have largely overlapping transcriptional functions, such as cell proliferation and cell migration; however, both have unique biological functions (Lai et al., 2018; Liu et al., 2018; Negron-Perez and Hansen, 2018). For example, YAP1 knockout (KO), rather than TAZ KO, in mice leads to embryonic death (Hossain et al., 2007; Makita et al., 2008). Conversely, parafibromin regulates the activity of YAP1 and TAZ in different status, which may explain the unique functions of YAP1 and TAZ.

Newly discovered co-transcription proteins reportedly contribute to YAP1/TAZ functions. Pyruvate kinase M2 (PKM2) is a relevant glycolytic protein; the interaction of YAP1 with hypoxia-inducible factor 1α (HIF-1α) promotes tumor cell glycolysis by triggering the transcription of the *Pkm2* gene (Zhang et al., 2018b). YAP1-mediated PKM2 expression enhances cell glycolysis and adapts tumor cells for abnormal growth. The SWI/SNF (Brg/Brahma-associated factors [BAF]) and ARID1A (BAF250A) proteins are inhibitors of the YAP1/TAZ-TEADs complex; in addition, these are capable of occupying the TEAD-binding site of YAP1/TAZ (Chang et al., 2018). Although the ARID1A–SWI/SNF complex is inactive during tumor growth, this negative association highlights the role of the ARID1A–SWI/SNF complex in cancer development through the suppression of oncogenic YAP1/TAZ activity (Kadoch and Crabtree, 2015).

Collectively, novel transcription mechanisms have been reported for explaining partly YAP1/TAZ biological output; these findings bode well for future YAP1/TAZ studies. However, the diversity of transcription partners and the target genes of YAP1/TAZ make it difficult to clarify the biological effects of YAP1/TAZ; therefore, future research should consider the entire molecular network of the YAP1/TAZ pathway.

## Regulation of the YAP1/TAZ by Post-Translational Modifications

### Regulation of the YAP1/TAZ by Phosphorylation

Yes-associated protein 1 and TAZ, two salient downstream effectors of Hippo signaling, are found both in the cytoplasm and nucleus. Since these two effectors trigger target gene transcription in the nucleus, nuclear accumulation of YAP1/TAZ is a key step in their mechanism of action. This nuclear accumulation is mediated by several events: (1) post-translational modifications (PTMs) including phosphorylation, methylation, and ubiquitination; (2) other related protein interactions with YAP1/TAZ; and (3) translational interference (Piccolo et al., 2014; Mo, 2017). In most scenarios, YAP1/TAZ are mediated by PTMs that determine their subcellular localization and content (Table 1).

**TABLE 1:** Post-translational modifications that modulate YAP1/TAZ.

Modification types	Regulative sites	Enzymes	Effects
Phosphorylation	Ser109	LATS1/2	YAP1/TAZ cytoplasm retention (Zhao et al., 2010)
	Ser127 (Ser89 in TAZ)	LATS1/2	Binding YAP1 to 14-3-3 and inducing cytoplasm retention (Zhao et al., 2007)
	Ser128	NLK	Blocking YAP1 binding to 14-3-3 (Moon et al., 2017)
	Ser381 (Ser311 in TAZ)	LATS1/2	β–Trcp-dependent ubiquitination degradation (Zhao et al., 2010)
	NS	PRP4K	YAP1/TAZ nuclear export (Cho et al., 2018)
	Tyr357	c-Abl	Stabilizing YAP1 stable and increasing YAP1–p73 interaction inducing cell apoptosis (Levy et al., 2008)
	Tyr357	Src	Enhancing YAP1 nuclear retention and target gene *CTGF* expression (Taniguchi et al., 2015)
	one or more tyrosine residues	Yes	Promoting YAP1 translocation and *Oct-3/4* transcription (Tamm et al., 2011)
Dephosphorylation	Ser127 (Ser89 and Ser311 in TAZ)	PP1A	YAP1/TAZ nuclear translocation (Liu et al., 2011; Wang et al., 2011).
O-GlcNacylation	Ser109	OGT	Impeding LATS1/2-induced YAP1 Ser109 phosphorylation and potentiating YAP1 activity (Peng et al., 2017)
	Thr241	OGT	Attenuating LATS1/2-dependent YAP1 Ser127 phosphorylation and enhancing YAP1 activity (Zhang et al., 2017)
Methylation	Lys342	SET1A	Dampening YAP1–CRM1 fusion and enhancing YAP1 nuclear retention (Fang et al., 2018)
	Lys494	SET7	Enhancing YAP1 translocation to cytosol and cytoplasmic membrane Oudhoff et al., 2013)
Ubiquitination	NS	SCF^β–TRCP^	YAP1/TAZ protein degradation (Dong et al., 2007; Zhao et al., 2007)
	NS	Fbxw7	YAP1 protein degradation (Tu et al., 2014; Zhang et al., 2016)
	Lys321, Lys497	SKP2	Accumulating YAP1 nuclear localization and association between YAP1 and TEAD (Yao et al., 2018)
De-ubiquitination	NS	USP9X	Stabilizing YAP1 and enhancing its activity (Li et al., 2018a).
	NS	OTUD1	Reducing YAP1 stability and attenuating the cell proliferation activity of YAP1 (Yao et al., 2018).

*NS, not specified, where the specific regulator sites have not been validated.*

In the canonical Hippo pathway, phosphorylation plays a pivotal role in the regulation of YAP1/TAZ; they can be phosphorylated at multiple sites, such as Ser61, Ser109, Ser127 (Ser89 in TAZ), Ser164, and Ser381 (Ser311 in TAZ) (Zhao et al., 2010). Among these, Ser127 and Ser381 are the main phosphorylation sites by LATS1/2. Following phosphorylation at Ser381, YAP1 is prone to further phosphorylation by the casein kinase I isoform δ/ε (CKI δ/ε), which eventually promotes SCF^β–TRCP^-induced ubiquitination degradation of YAP1 (Zhao et al., 2010). YAP1 binds to 14-3-3 protein through the Ser127 phosphorylation-dependent way and becomes sequestered in the cytoplasm (Zhao et al., 2007). Notably, the combination of YAP1 and 14-3-3 can be blocked by phosphorylation at the YAP1 Ser128 site by Nemo-like kinase (NLK); this phenomenon can also be seen in *Drosophila* Nemo (Moon et al., 2017). This indicates that NLK/Nemo are highly conserved in Hippo-YAP1/TAZ signaling to mediate the YAP1/Yki subcellular compartmentation via phosphorylation.

Phosphorylation-dependent cytoplasmic retention of YAP1/TAZ may hamper their transcriptional activity. In the transgenic heart, MST1 overexpression was shown to enhance the phosphorylation of YAP1 Ser127 and induce inactivation of YAP1; this induced cardiomyocyte (CM) apoptosis and myocardial fibrosis (Yamamoto et al., 2003; Zhao et al., 2019). In LATS1/2 cardiac knock out (CKO) of the embryonic epicardium, LAST1/2 deficiency was shown to inhibit the phosphorylation of YAP1 Ser127 and increase the YAP1 transcriptional activity; eventually, this resulted in the failure of subepicardial cells to differentiate into cardiac fibroblasts and induced coronary vessel defects (Xiao et al., 2018). Thus, the canonical Hippo pathway inhibits the YAP1/TAZ activity in cardiac tissues through phosphorylation. Interestingly, YAP1/TAZ was shown to increase the concentration and activity of endogenous LATS2 by directly inducing TEAD-dependent *LATS2* transcription and/or indirectly activating neurofibromin 2 (NF2)-mediated LATS2 activity, which subsequently enhances the phosphorylation of YAP1/TAZ (Moroishi et al., 2015; Dai et al., 2017). This physiological negative feedback mechanism is expected to maintain the dynamic homeostasis of the Hippo pathway, and may help elucidate the complex mechanism of cardiac development and disease.

Notably, YAP1/TAZ can be dephosphorylated by a catalytic subunit of protein phosphatase-1 (PP1A) at Ser127 (Ser89 and Ser311 in TAZ), and is then translocated into the nucleus, thereby causing leak activation of the target gene (Liu et al., 2011; Wang et al., 2011). This abnormal activation can be potentially prevented by some mechanisms. The pre-mRNA splicing factor 4 kinase (PRP4K) promotes phosphorylation of Yki/YAP1/TAZ in the nucleus as well as its subsequent nuclear export; this acts as a safety threshold for the rescue of aberrant Yki/YAP1/TAZ activities and cell growth (Cho et al., 2018).

Besides serine phosphorylation, the subcellular distribution and activities of YAP1/TAZ are affected by the phosphorylation of tyrosine residues. Several members of the SRC family of kinases (SFK), such as Yes, Src, and c-Abl, participate in tyrosine phosphorylation of YAP1. In response to DNA damage, YAP1 Tyr357 phosphorylation by c-Abl stabilizes YAP1 and increases the nuclear interaction of YAP1-p73 to induce apoptosis (Levy et al., 2008). This phosphorylation may inhibit the cell proliferation and oncogenic ability of YAP1 that are necessary to sustain its normal biological functions. The activated Yes phosphorylates YAP1 on one or more tyrosine residues; the phosphorylated YAP1 is subsequently translocated to the nucleus to trigger the transcription of the pluripotency factor *Oct-3/4*, which is critical for maintaining the self-renewal ability of the mouse and human embryonic stem cells (hESCs) (Tamm et al., 2011). Furthermore, Src phosphorylated by the IL6–GP130 complex phosphorylates YAP1 at Tyr357, which increases YAP1 nuclear retention and expression of its target gene *connective tissue growth factor* (*CTGF*); this Src-induced YAP phosphorylation facilitates the regeneration of the injured intestinal mucosa (Taniguchi et al., 2015). The above-mentioned research shows that tyrosine phosphorylation participates in the regulation of YAP1/TAZ in specific cell and tissue regeneration. This suggests that the tyrosine phosphorylation of YAP1/TAZ may be conductive to embryonic cardiac development and post-injury cardiac regeneration; these findings provide new insights into cardiac development and regeneration.

### Regulation of the YAP1/TAZ by O-GlcNAcylation

Besides phosphorylation, several PTMs, such as O-GlcNAcylation, are involved in the regulation of YAP1/TAZ. O-GlcNAc transferase (OGT) directly induces O-GlcNAcylation at YAP1 Ser109 and Thr241, and potentiates the pro-proliferation activity of YAP1 in pancreatic cancer and liver cancer cells (Peng et al., 2017; Zhang et al., 2017). Mechanistically, YAP1 O-GlcNAcylation can affect YAP1 phosphorylation. As mentioned in Section “Regulation of the YAP1/TAZ by Phosphorylation,” LATS1/2 can phosphorylate YAP1 Ser109 (Zhao et al., 2010). Therefore, O-GlcNAcylation and phosphorylation may act competitively at Ser109. The stoichiometry of Ser109 phosphorylation is much lower than that of O-GlcNAcylation, which suggests that O-GlcNAcylation plays a key role in PTM at this site rather than phosphorylation (Peng et al., 2017). O-GlcNAcylation at YAP1 Thr241 can attenuate the phosphorylation of YAP1 at Ser127 via interrupting the LATS1/2 interaction (Zhang et al., 2017). In addition, these studies reveal that YAP1 not only acts as a substrate of OGT but also triggers *OGT* transcription to regulate its expression, thereby forming a positive feedback loop (Peng et al., 2017; Zhang et al., 2017). This positive feedback loop further regulates YAP1 activity and output.

Therefore, YAP1 may participate in cancer glycosylation metabolism through O-GlcNAcylation and OGT expression in order to maintain the abnormal proliferation and survival of cancer cells. *In vitro*, OGT KO inhibits O-GlcNAcylation at the YAP1 Ser109, thereby inhibiting cell-colony formation in pancreatic cancer; *in vivo*, OGT knockdown downregulates tumor growth (Peng et al., 2017). High glucose stimulation improves the level of O-GlcNAcylation at YAP1 Thr241; in addition, O-GlcNAcylation enhances the stability and activity of YAP1, which subsequently induces cell proliferation and transformative phenotypes in hepatocellular carcinoma cells (HCC, THLE-3, and HL-7702 cell lines) (Zhang et al., 2017). In addition to liver cancer, high glucose level affects diabetic cardiomyopathy. Elevated cardiac concentrations of active YAP1 have been demonstrated in mice fed with high-fat diet and in patients with diabetic cardiomyopathy (Ikeda et al., 2019b). However, the relationship between YAP1 and diabetic cardiomyopathy is not completely clear. Thus, YAP1 O-GlcNAcylation might be a promising target for the study of diabetic cardiomyopathy.

### Regulation of the YAP1/TAZ by Ubiquitination

The stability and content of YAP1/TAZ are partly regulated by ubiquitination-induced degradation; therefore, ubiquitination cannot be neglected in research into YAP1/TAZ. SCF^β–TRCP^-dependent ubiquitination is a well-known YAP1/TAZ degradation mechanism in the canonical Hippo pathway that is dependent on YAP1/TAZ phosphorylation (Dong et al., 2007; Zhao et al., 2007). Similarly, Fbxw7, another subunit of E3 ligase, participates in the regulation of YAP1 protein level and activity via ubiquitination and proteasomal degradation (Tu et al., 2014; Zhang et al., 2016). Fbxw7 expression exhibits a negative correlation with YAP1 content in human HCC and pancreatic ductal adenocarcinoma tissues; however, the regulatory mechanisms are yet to be elucidated. This proteolytic ubiquitination can be reversed by deubiquitinases; the deubiquitinase USP9X was shown to directly deubiquitinate and stabilize YAP1, leading to enhanced YAP1 activity and promotion of tumor growth (Li et al., 2018a). LATS1/2, activated by mono-ubiquitinated angiomotin-like 2 (AMOTL2), triggers YAP1 phosphorylation and suppresses its activity (Kim et al., 2016). USP9X deubiquitinates AMOTL2 to inactivate LATS1/2, which indirectly activates YAP1 (Kim et al., 2016). Therefore, ubiquitination appears to be a negative regulatory factor for YAP1/TAZ in the canonical Hippo pathway.

Intriguingly, recent studies have provided new insights into ubiquitination independent of the Hippo pathway. The SCF^SKP2^ E3 ligase complex (SKP2) facilitates K63-linkage-specific ubiquitination of YAP1 at Lys321 and Lys497, which does not reduce YAP1 abundance (Yao et al., 2018). Moreover, these ubiquitination promotes nuclear translocation of YAP1, and cements the association between YAP1 and TEAD to enhance the cell proliferation activity of YAP1 (Yao et al., 2018). This non-proteolytic ubiquitination can be reversed by the binding and interaction between deubiquitinase OTUD1 and YAP1; OTUD1 decreases YAP1 stability, and attenuates the cell proliferation and tumor-growth activity of YAP1 (Yao et al., 2018). Obviously, this non-proteolytic ubiquitination is beneficial for YAP1 activation and output. The above-mentioned studies delineate the controversial role of ubiquitination in YAP1/TAZ in the canonical Hippo–YAP1/TAZ pathway as well as in the Hippo-independent pathway. YAP1/TAZ is degraded by ubiquitinases that exert proteolytic effects, whereas it is stabilized by ubiquitinases with non-proteolytic activities; these events can be reversed by the related deubiquitinases. Undoubtedly, ubiquitination plays an essential role in mediating YAP1/TAZ content and stability that should be further studied. The biological effects of ubiquitinated/deubiquitinated YAP1/TAZ have been investigated mainly in tumorigenesis, and less so in cardiogenesis and cardiac disease.

### Regulation of the YAP1/TAZ by Methylation

Chromosomal maintenance 1 (CRM1), a nuclear export protein, triggers the nuclear export of YAP1. The methyltransferase SET1A targets YAP1 at Lys342 through mono-methylation, which inhibits CRM1-induced nuclear export of YAP1 with resultant enhancement of YAP1 nuclear retention and transcriptional activity (Fang et al., 2018). Mono-methylation at Lys342 in YAP1 contributes to *in vivo* cell proliferation and tumorigenesis (Fang et al., 2018). However, this result was discordant with that from a previous study on mono-methylated YAP1. The SET7 is a methyltransferase that connects the cell membrane and the cytoskeleton (Garbino et al., 2009). Mono-methylation of YAP1 Lys494 by SET7 is necessary for the retention of YAP1 in the cytosol and at the cytoplasmic membrane, which may impede interactions between YAP1 and PDZ-dependent-binding partners (Oudhoff et al., 2013). Thus, methylation at YAP1 Lys494 represses YAP1 activity to downregulate *CTGF* transcription. Evidently, methylation can exert certain effects on YAP1/TAZ, but there is a paucity of studies on YAP1/TAZ methylation and their biological output. In addition, the mechanisms by which methylated YAP1/TAZ affect cardiac biology remain unknown.

Several types of PTMs alter the subcellular translocation of YAP1/TAZ to regulate their activities and output, regardless of how YAP1/TAZ are modified by PTMs. Therefore, it is important to study the nuclear–cytoplasmic trafficking of YAP1/TAZ. An increasing body of evidence supports the mechanisms of YAP1/TAZ nucleocytoplasmic shuttling. In a stiff cultured environment, the nucleus encounters mechanical forces and becomes flattened; in addition, the nuclear pores are stretched, subsequently creating conditions for nuclear import of YAP1 (Elosegui-Artola et al., 2017). A nuclear localization sequence (NLS) and nuclear export sequence (NES) were identified within TAZ, which contributes to the nucleocytoplasmic distribution of TAZ in different ways: TEAD binding interaction partly conceals the NES; RhoA enhances NLS-induced nuclear import; and TEAD and 14-3-3 competitively bind with TAZ (Kofler et al., 2018).

Collectively, PTMs can alter YAP1/TAZ stability and/or YAP1/TAZ conformation to change its affinity for other proteins; these eventually mediate YAP1/TAZ subcellular location and content. PTMs mediate YAP1/TAZ through interdependent, synergistic, or competitive mechanisms. A growing body of evidence has revealed the underlying mechanisms of PTMs and YAP1/TAZ activity; however, several important nuances are yet to be elucidated.

## Hippo–YAP1/TAZ Signaling in Cardiac Development

Cardiogenesis involves events that alter the spatiotemporal and morphological mechanisms. In the gastrulation period, the mesoderm, which emanates from the anterior primitive streak (APS), produces cardiac progenitor cells (Tam et al., 1997). Subsequently, cardiac progenitor cells form two different heart fields: the primary heart field gives rise to the left ventricle and left/right atria, while the secondary heart field generates the right ventricle, left/right atria, and the outflow tract (Xin et al., 2013b). Postnatally, there is a decrease in CM proliferation alongside an increase in hypertrophy which helps attain the physiologic cardiac size. At approximately postnatal Day 4 (P4), myocardial cell numbers peak, and then stop increasing (Li et al., 1996). Myocardial proliferation can be blunted as early as E11.5 with the help of cardiac gene reprogram; this results in abnormal cardiac growth and perinatal lethality (Wang et al., 2014).

### Role of Hippo–YAP1/TAZ Signaling and Its Proliferation Output in Heart Development

The Hippo pathway is a highly conserved mechanism for the regulation of heart size and maturation (Harvey et al., 2003; Pan, 2007; Hayashi et al., 2015). Its downstream transcriptional factors, YAP1/TAZ, are key regulators of embryonic and neonatal cardiac development, and this has been validated by multiple lines of evidence (Tables 2, 3). Mouse embryonic heart with SAV1 CKO (*Nkx2.5^cre^*: *SAV*^f/f^) shows ventricular septal defect and abnormal heart growth, including thickening of ventricular walls, expansion of trabecular and ventricular myocardial layers, and enlargement of ventricular chambers (Heallen et al., 2011). Similarly, LATS2 and MST1/2 CKO hearts show the same abnormal heart phenotypes (Matsui et al., 2008; Heallen et al., 2011). The SAV CKO heart with reduced phosphorylation of YAP1 exhibit enhanced proliferation of CM and unchanged cell size (Heallen et al., 2011). A WWTR1 (or TAZ) deficiency in zebrafish heart results in arrested CM proliferation, reduced cardiac trabeculation, and immature trabecular bridges (Lai et al., 2018). YAP1 knockdown using Tnnt2-Cre in mouse fetal heart causes cardiac hypoplasia with reduced ventricular chamber size, ventricular septal defects, peripheral edema, and pericardial effusion; these embryos cannot survive beyond E16.5 (von Gise et al., 2012). YAP1 inactivation represses fetal CM proliferation; however, it does not affect the cell size. Consistent with this, YAP1 deletion in the postnatal heart using Tnnt2-Cre does not significantly affect physical cardiac hypertrophy (von Gise et al., 2012). Thus, the Hippo pathway regulates the normal cardiac structure and size by mediating the cell proliferation activities of YAP1/TAZ in the fetal and postnatal phase.

**TABLE 2:** Cardiac output of the Hippo–YAP1/TAZ pathway in cell and animal models.

Gene	Model		Promoter	Output
YAP1	Mouse	CKO	Tnnt2-Cre	Cardiac hypoplasia (reduced ventricular chamber size, ventricular septal defects, peripheral edema, and pericardial effusion) (von Gise et al., 2012)
	Mouse	CKO	Nfatc1^IRES–Cre/+^	Less CM proliferation, impaired compact myocardium, and early postnatal lethality (Artap et al., 2018)
	Mouse	CKO	α-MHC-Cre	Blunted cardiac hypertrophy and amplified CM apoptosis and fibrosis; cardiac dilatation and dysfunction after TAC (Byun et al., 2019)
	Mouse	CKO	Nkx2.5-cre	Lack of healthy myocardial tissue in the left ventricle wall and an enhancing fibrotic infarct zone following MI (Xin et al., 2013a)
	Mouse	CKO	α-MHC-Cre	Thinned septal and posterior wall, and chamber dilation (Del Re et al., 2013).
	Mouse	CKO	α-MHC-Cre	Increasing CM apoptosis, fibrosis, enlarging infarct size, and impairing cardiac function (Del Re et al., 2013)
	Mouse	Overexpression	Adeno-associated virus subtype 9: human YAP1	Alleviating MI injury and ameliorating cardiac function (Lin et al., 2014)
	Mouse	YAP5SA (active YAP1) overexpression	αMyHC-Cre-ERT2	Re-entering the cell cycle and reprogramming into more primitive and fetal cell states; thickened ventricular walls and smaller chambers (Monroe et al., 2019)
	Mouse	YAPS112A (active YAP1) overexpression	α-MHC	Increased myocardial tissue and reduced LV fibrosis in neonatal heart (Xin et al., 2013a)
	AC16 human CMs	Overexpression	Lentiviral vectors	Reducing CM apoptosis, cell hypertrophy, and ROS generation after IR (Khan et al., 2019)
WWTR1 (TAZ)	Zebrafish	CKO	CRISPR/CAS9	CM proliferation arrest, reduced cardiac trabeculation, and immature trabecular bridges (Lai et al., 2018)
WW45	Mouse	CKO	Myh6-Cre	Sustained YAP1 activation in CMs with cell-cycle re-entry, increased de-differentiation, and decreased apoptosis; cardiac dysfunction, severe heart failure, and enhanced mortality in response to TAC (Ikeda et al., 2019a, b)
SAV1	Mouse	CKO	Nkx2.5-cre	Ventricular septal defect and abnormal heart growth (thickening of ventricular walls, expansion of trabecular and ventricular myocardial layers, and enlargement of ventricular chambers) (Heallen et al., 2011)
	Mouse	CKO	Myh6^CreERT2^ αMHC-mcm	Presenting renewal capacity: increase in cell number and myocardial regeneration following cardiac apex resection in postnatal hearts; increased LV CMs, less fibrosis, and improved cardiac function after MI in adult heart (Heallen et al., 2013; Leach et al., 2017)
RASSF1A	Mouse	cardiomyocyte-specific KO	α-MHC-Cre	Basal nondistinctive cardiac phenotype or functional abnormality; reduced apoptosis, fibrosis, and hypertrophy after TAC (Del Re et al., 2010)
	Mouse	KO	NS	Increased hypertrophic response, reduced cardiomyocytes apoptosis, and increased fibrosis after TAC (Del Re et al., 2010)
MST1/2	Mouse	CKO	Nkx2.5-cre	Ventricular septal defect and abnormal heart growth (Heallen et al., 2011)
MST1	Mouse	DN-MST1 overexpression	CMV-Cre	Reducing the size of MI in the area at risk, and decreasing CM apoptosis (Nakamura et al., 2016)
	Mouse	DN-MST1 overexpression	α-MHC	Reduced left ventricular remodeling, improved left ventricular function, and enhanced survival rate after MI (Maejima et al., 2013)
LATS2	Mouse	LATS2 overexpression	α-MHC	Reduced left ventricular systolic and diastolic dysfunction, and smaller left/right ventricle (Matsui et al., 2008)
	Mouse	DN-LATS2 overexpression	α-MHC	Reduced CM apoptosis and enhanced biventricular hypertrophy following TAC (Matsui et al., 2008)
	Mouse	CKO	Nkx2.5-cre	Ventricular septal defect and abnormal heart growth (Heallen et al., 2011)
LATS1/2	Mouse	LATS1/2 CKO; YAP1/TAZ CKO	Wt^CreERT2^ allele	Successful survival past E15.5 without defects in coronary vasculature (Xiao et al., 2018)
	Mouse	CKO	Wt^CreERT2^ allele	Failing to survive past E15.5; smaller hearts with less compacted myocardium (Xiao et al., 2018)
	Mouse	CKO	Myh6^CreERT2^	Presenting renewal capacity: increasing cell number and regenerating the myocardium following cardiac apex resection in postnatal hearts; increased LV CMs, less fibrosis, and improved cardiac function after MI in adult heart (Heallen et al., 2013)
	Zebrafish	DKO	Myh6; Myl7	Enhanced Hand2 expression and CM differentiation (Schindler et al., 2014; Fukui et al., 2018)

*NS, not specified, where the specific promotors have not been validated; YAP1, Yes-associated protein 1; TAZ, PDZ-binding motif; CKO, cardiac knockout; KO, knockout; DN, dominant negative, MI, myocardial infarction; CM, cardiomyocytes; TAC, transverse aortic constriction; IR, ischemia–reperfusion; ROS, reactive oxygen species.*

**TABLE 3:** Target genes and cardiac output of the Hippo–YAP1/TAZ pathway.

Target genes	Type of Regulation	Outcome
*Bmp2b*	Promotion	Activating the BMP pathway to enhance the number of CPCs in the secondary heart field (Fukui et al., 2018)
*Cyr61* and *CTGF*	Promotion	Promoting CM proliferation (Badouel et al., 2015; Ragni et al., 2017; Zou et al., 2018)
*Dpp4* and *Dhrs3*	Promotion	Inhibiting subepicardial cells from differentiating into cardiac fibroblasts, thus inducing coronary vessel defects (Xiao et al., 2018)
*hand2*	Promotion	Regulating differentiation of LPM cells that develop into the atrium of the heart (Fukui et al., 2018)
*miR-152*	Promotion	Promoting CM proliferation (Wang et al., 2018)
*miR-206*	Promotion	Promoting cell hypertrophy and cell survival by downregulating FoxP1 after TAC (Yang et al., 2015)
*Nrg1*	Promotion	Activating Nrg1/ErbB2 signaling and reducing CM proliferation, to impair compact myocardium development (Artap et al., 2018).
*Oct-3/4*	Promotion	Maintaining mouse and human embryonic stem cell self-renewal (Tamm et al., 2011)
*OSM*	Promotion	Regulating CM de-differentiation (Ikeda et al., 2019a)
*Park2*	Promotion	Promoting the clearance of impaired mitochondria through autophagy, subsequently enhancing CM resistance to stress (Kubli et al., 2013; Leach et al., 2017).
*Pik3cb*	Promotion	Activating PI3K-AKT signaling to trigger CM proliferation and survival (Xin et al., 2011; Lin et al., 2015)
*Sox17*	Inhibition	Disrupting CM differentiation (Hsu et al., 2018)

*CM, cardiomyocytes; PI3K, phosphoinositol 3-kinase; TAC, transverse aortic constriction; LPM, lateral-plate mesoderm, CPC, cardiac precursor cell.*

Yes-associated protein 1/TAZ, as vital effectors of the Hippo pathway, mainly affect target genes transcription and downstream signaling to exhibit their pro-proliferation activities in heart development (Table 3). Neuregulin 1 (Nrg1), secreted by endocardial cells, is a crucial factor in myocardial growth; it induces CM proliferation and re-entry of differentiated CMs in the cell-cycle via Nrg1/ErbB2 signaling (Bersell et al., 2009; Gemberling et al., 2015). Targeting at *Nrg1*, YAP1/TAZ activates Nrg1/ErbB2 signaling in the endocardium; in addition, loss of YAP1 in the endocardium contributes to less CM proliferation, impaired development of compact myocardium, and early postnatal mortality (Artap et al., 2018). Moreover, Nrg1/ErbB4 signaling can reduce the phosphorylation of YAP1 Ser127, and promote YAP1-dependent *CTGF* transcription through the production of the ErbB4–YAP1–TEAD complex in breast cancer cells (Haskins et al., 2014). Thus, it is hypothesized that YAP1 proliferatively increases output not only by directly mediating *CTGF* transcription, but also indirectly by activating Nrg1/ErbB4 signaling. However, it is unclear whether Nrg1/ErbB4 signaling-dependent YAP1 activation exists in the endocardium.

Through differential gene expression analysis, *Pik3cb* [which encodes a catalytic subunit of phosphoinositol-3-kinase (PI3K)] has been identified as the target gene of YAP1. YAP1 promotes *Pik3cb* transcription to activate PI3K-AKT signaling and trigger CM proliferation and survival (Xin et al., 2011; Lin et al., 2015). In cases with Pik3cb deficiency, the mouse embryo cannot survive beyond E10.5 (Bi et al., 2002). Moreover, PI3K-AKT signaling inhibits glycogen synthase kinase 3β (GSK3β)-dependent phosphorylation of β-catenin; the consequent activation of β-catenin promotes CM proliferation (Xin et al., 2011). The interaction between the Hippo pathway, Wnt/β-catenin signaling, and PI3K-AKT signaling facilitates the proliferation of CMs. Besides the aforementioned signaling, YAP1/TAZ can also affect bone morphogenetic protein (BMP) pathway to regulate CM proliferation. By upregulating *Bmp2b* transcription, YAP1/TAZ activate the BMP pathway to enhance the number of cardiac precursor cells (CPCs) in the secondary cardiac field (Fukui et al., 2018). More importantly, BMP2 induces EMT in cardiac cushion, which facilitates the development of valves and septa (Gomez-Puerto et al., 2019). These mechanisms delineate the crosstalk between YAP1/TAZ and BMP pathway in cardiac development. Collectively, the above-mentioned findings suggest that the pro-proliferation activities of YAP1/TAZ participate in cardiac development from the fetal to postnatal periods, including the trabeculation and formation of the ventricles and endocardium. Thus, the functions of the Hippo pathway and YAP1/TAZ are shown to be highly conserved, as is the crosstalk between the Hippo pathway and other signaling pathways in cardiac development.

### Role of Hippo–YAP1/TAZ Signaling and Its Differentiation Output in Heart Development

Besides cell proliferation, cardiogenesis involves cell differentiation; in addition, different cell types are involved in salient events during cardiac development. YAP1/TAZ are the key inhibitors of hESC differentiation into cardiac mesoderm. The CKO of LATS1/2 expression in the epicardium by using Wt^CreERT2^ prevents embryos from surviving past E15.5 (Xiao et al., 2018). Due to the deletion of LATS1/2, activated YAP1 inhibits the differentiation of subepicardial cells into cardiac fibroblasts which induces coronary vascular defects; however, the LATS1/2 CKO and YAP1/TAZ CKO embryos do not exhibit defects of the coronary vasculature. This indicates that LATS1/2 plays an important role in restricting YAP1/TAZ activities and that YAP1/TAZ are essential for normal heart growth (Xiao et al., 2018). In LATS1/2 CKO heart, downstream genes *Dpp4* and *Dhrs3* transcription is upregulated through the YAP1-TEAD complex, which regulates the differentiation events of subepicardial cells and the coronary vessel remodeling, respectively (Xiao et al., 2018). Dehydrogenase/reductase superfamily 3 (Dhrs3), a negative modulator of retinoic acid generation, inhibits retinoic acid signaling, which inhibits the differentiation of cardiac fibroblasts (Billings et al., 2013; Xiao et al., 2018). In response to LATS1/2 deficiency, Dipeptidyl peptidase-4 (Dpp4; a serine protease) alters the composition of the ECM and is involved in abnormal vessel development, such as increasing blood islands and defects of mean lacunarity (Ghersi et al., 2006; Xiao et al., 2018).

Furthermore, YAP1/TAZ regulate the transcription of other target genes to affect the progression of cell differentiation in cardiogenesis, such as *Sox17* and *hand2*. *Sox17*, a member of *SOX* genes, plays a critical role in cardiac development. YAP1 selectively represses the expression of APS cell genes *Sox17* to disrupt the differentiation of CMs (Hsu et al., 2018). Inhibition of YAP1 by dasatinib induces the differentiation of hESCs into APS-derived endoderm and cardiac mesoderm. In addition to cardiac mesoderm, Sox17 is also required for the development of endocardium. Sox17 deficiency leads to abnormal endocardium with impaired ventricular trabeculation and thickened myocardium (Lange et al., 2014). YAP1/TAZ, through facilitation of *hand2* transcription, can regulate the differentiation of the lateral-plate mesoderm (LPM) cells, which finally develop into the atrium of the heart (Fukui et al., 2018). Hand2 can facilitate CM differentiation, and the expression of hand2 can be potentiated by LATS1/2 double KO (DKO) (Schindler et al., 2014; Fukui et al., 2018). Therefore, YAP1/TAZ can facilitate de-differentiation and differentiation activity in different cell types during various phases of cardiac development.

### Regulation of YAP1/TAZ by Diverse Mechanisms in Heart Development

During cardiac development, the content and activity of YAP1/TAZ are not always in a stable level. At approximately P4, myocardial cell numbers peak and this increase is reconciled with the percentage of nuclear YAP1 (Li et al., 1996). During the late maturation of trabeculae, CMs need to withdraw from the cell cycle and differentiate; if not, non-compaction cardiomyopathy occurs. YAP1 may modulate these events (Hertig et al., 2018). These studies indicate that during the early stage of development, there are high levels of YAP1/TAZ activity and abundance, contributing to cell proliferation and de-differentiation; however, in the late stage, the proliferation effects of YAP1/TAZ are blunted for normal cardiac development, which underscores the importance of the spatiotemporal modulation of YAP1/TAZ. Thus, YAP1/TAZ activities need to be fine-tuned spatiotemporally depending upon the pace of cardiogenesis.

A diverse range of mechanisms are involved in the precise modulation of YAP1/TAZ activities in cardiac homeostasis and appropriate cardiac size. As noted previously, it is imperative to focus on the PTMs of YAP1/TAZ, as these affect the subcellular localization and activity of YAP1/TAZ. Previous studies have mainly shown that the phosphorylation of YAP1/TAZ is dependent on the canonical Hippo pathway. In the SAV1 CKO heart, YAP1 that is phosphorylated at Ser127 is reduced, thereby causing CM proliferation (Heallen et al., 2011). Moreover, Nrg1/ErbB4 signaling can inhibit LATS1-dependent phosphorylation at YAP1 Ser127 and promote YAP1-dependent *CTGF* transcription (Haskins et al., 2014). Cullin-RING ligases (CRLs), also known as the ubiquitin ligases, consist of Cullins, substrate-recognition, and RING proteins that mediate proteolysis of cellular proteins (Petroski and Deshaies, 2005). CRL-dependent ubiquitination appears to play an important role in temporal regulation of the Hippo–YAP1/TAZ signaling during cardiac development. In the developing heart, CRLs trigger the ubiquitination-induced degradation of MST1; this, in turn, induces dephosphorylation and nuclear translocation of YAP1, thereby ensuring CM proliferation (Zou et al., 2018). Nonetheless, the ubiquitination effect of CRLs depends on the neddylation of a ubiquitin-like protein, NEDD8. Consequently, cardiac-specific inhibition of NEDD8 attenuates the activity of CRLs and thereafter blunts the cell-proliferation activity of YAP1; this causes ventricular hypoplasia and non-compaction and, eventually, heart failure and neonatal death in mice (Zou et al., 2018).

In the developing heart, endogenous microRNAs (miRNAs) regulate the Hippo pathway and YAP1/TAZ to maintain heart homeostasis; miRNAs, small non-coding RNA molecules, silence mRNAs and suppress mRNA translation to control cardiac development (Ambros, 2004; Bartel, 2004). miR302-367 restrains MST1, LATS2, and MOB1b, which reduces the phosphorylation of YAP1 Ser127 and enhances the nuclear translocation of YAP1 (Tian et al., 2015). miR302-367, through activation of YAP1, promotes cell proliferation in embryonic and postnatal CMs (Tian et al., 2015). Using functional screen and computational approaches, some miRNAs were found to promote YAP1-dependent CM proliferation by targeting and inhibiting the members of Hippo signaling (Tian et al., 2015; Diez-Cunado et al., 2018). For example, the predicted targets of miR-520d-3p are TAOK1/2 and LATS2; in addition, members of the miR-17 family repress TAOK1/2/3, MST2, SAV1, LATS2, MOBKL1A, and others (Diez-Cunado et al., 2018). Subsequently, miR-520d-3p, miR-17 family and other detected miRNAs enhance the nuclear localization of YAP1 and induce pro-proliferation activity. However, inactivation of a single miRNA (such as miR-520d-3p or miR-17 family) does not have any obvious effect on YAP1 activity; this suggests that none of these miRNAs can individually maintain YAP1 nuclear location and cell proliferation. This indicates that multiple endogenous miRNAs synergistically, but not individually, regulate Hippo signaling-induced cell proliferation during cardiac development.

In the early postnatal period, cell–cell junctions in CMs progressively mature and modulate nuclear-cytoplasmic localization of YAP1/TAZ. The dystrophin-glycoprotein complex (DGC), a kind of transmembrane complex, serves as a link between the cellular cytoskeletal system and the ECM (Srivastava and Yu, 2006). DGC directly binds to YAP1 at the cell membrane, suppressing YAP1 activity. This interaction is synergistically augmented by the activation of the Hippo pathway; this suggests that Hippo signaling and DGC work in concert to inhibit the proliferation of murine CM (Morikawa et al., 2017). A cell junction protein Fat4 binds angiomotin-like 1 (AMOTL1, a isoform of angiomotin [AMOT] family) with YAP1 at the cell junction, leading to impaired cell proliferation in postnatal mouse heart; this is independent of the canonical Hippo pathway (Ragni et al., 2017). In the absence of Fat4, YAP1 translocates to the nucleus, promoting YAP1-dependent *CTGF* transcription; this leads to thicker ventricular myocardium and interventricular septum in the mouse heart with increased cell proliferation (Badouel et al., 2015; Ragni et al., 2017). Similar to this, AMOTL1 can bind to LATS2 at tight junction, and activate LATS2 to enhance YAP1 phosphorylation and repress YAP1 activity (Paramasivam et al., 2011). Therefore, cell junction related proteins may induce cytoplasmic sequestration of YAP1/TAZ; the consequent inhibition of YAP1 activity impairs heart growth after birth. Interestingly, AMOTL1 exhibits a dichotomous effect on the regulation of YAP1 activity. YAP1, forming a complex with AMOTL1, co-translocates to the nucleus and triggers proliferation activity as a result of Fat4 deficiency (Ragni et al., 2017); in addition, AMOTL1 inhibits YAP1 with the help of other junction proteins or the Hippo pathway (Paramasivam et al., 2011).

The above-mentioned studies reveal that the protein level and stability of YAP1/TAZ play a pivotal role in cardiac function and development. YAP1/YAZ imbalance leads to abnormal cardiac development, dysfunction, and even mortality. And YAP1/TAZ are mediated by several complex mechanisms such as PTMs, endogenous miRNAs, and cell–cell junctions, which makes it difficult to understand the complete molecular network and remains to be studied.

## Role of Hippo–YAP1/TAZ Signaling in Cardiac Disease

The heart can be damaged by several stimuli such as inadequate nutrition, oxygen deficiency, and hemodynamic stress. Among these, ischemic heart disease plays a major role in cardiac disease. The regulation of the Hippo–YAP1/TAZ pathway appears to be cardiac protective, with relevant effects on cell proliferation, apoptosis, and cardiac remodeling (Tables 2, 3).

### Role of Hippo–YAP1/TAZ Signaling in Cardiac Ischemia-Reperfusion and Myocardial Infarction

An increasing body of evidence has implicated YAP1/TAZ in the causation of cardiac ischemia-reperfusion (IR) damage. IR induces activation of MST1 in mouse heart; however, after overexpressing dominant-negative MST1 (DN-MST1), the inactive MST1 induces reduction in myocardial infarction (MI) size in areas at risk of mouse heart (Nakamura et al., 2016). Endogenous MST1 potentiates IR-induced myocardial injury mainly by increasing apoptosis and partly by inhibiting autophagy, which is independent of the canonical Hippo pathway (Yamamoto et al., 2003; Nakamura et al., 2016). Mechanistically, the Ras-association domain family 1 isoform A (RASSF1A) enhances MST1 activity and translocation to the mitochondria; thereafter, activated MTS1 augments Bax- or Bcl-xL-induced apoptosis and suppresses Beclin1-mediated autophagy (Yamamoto et al., 2003; Nakamura et al., 2016). In this setting, CM autophagy, as a protective mechanism, degrades and recycles harmful cytoplasmic components, which contributes to repair of IR damage and promotes cell survival; in contrast, apoptosis was shown to perform an anti-survival role in IR damage (Nishida et al., 2008). Similar results have been observed in models of MI. After MI, endogenous MST1 potentiates left ventricular (LV) enlargement, impairs LV ejection fraction, and reduces the survival rate by suppressing autophagy (Odashima et al., 2007; Maejima et al., 2013). MI-mediated MST1 activation stabilizes the Beclin1 homodimer, and suppresses the downstream output of Beclin1 to inhibit autophagy; this impairs the recycling of harmful components and protein quality control (Maejima et al., 2013). Besides the MST1–Bax–Bcl-xL axis, MST1 mediates the canonical Hippo pathway to exert pro-apoptotic activity. In cultured CMs, LATS2 was shown to increase cell apoptosis in a dose-dependent manner; in contrast, dominant-negative LATS2 was found to suppress MST1-induced apoptosis (Matsui et al., 2008). IR induces activation of NF2 and potentiation of MST1 activity, which increases CM apoptosis; hearts lacking NF2 by Cre^αMHC^ exhibit diminished phosphorylation of MST1 and activation of YAP1 (Matsuda et al., 2016). In the NF2 CKO heart, enhanced YAP1 activity protects against IR damage. Collectively, these findings suggest that MST1 participates in IR and MI injury by enhancing apoptosis and reducing autophagy; moreover, MST1 works as a negative regulator in cardiac protection through Hippo-dependent or -independent mechanisms. Based on the available evidence, it can be indirectly inferred that the Hippo pathway may be an anti-survival mediator in cardiac IR and MI damage.

As mentioned previously, IR and MI stress induce activation of the Hippo pathway and inactivation of YAP1/TAZ. Activated YAP1 has a protective effect on IR-damaged heart. Lentivirus-generated YAP1 overexpression in AC16 human CMs decreases CM apoptosis, cell hypertrophy, and generation of reactive oxygen species (ROS), which protects CMs from IR injury (Khan et al., 2019). Interestingly, downregulation of the hypertrophy effect of YAP1 is not consistent with the results of previous studies that showed that YAP1 promotes CM hypertrophy after IR stress (Yang et al., 2015; Zhou et al., 2015). This downregulation of the hypertrophy effect may be secondary to decreased apoptosis and ROS generation instead of direct regulation by YAP1; therefore, there exists a difference. Several mechanisms explain the cardioprotective function of YAP1. YAP1 inhibits the ataxia–telangiectasia mutated (ATM)/ATM-and Rad3-related (ATR) DNA-damage cascade to decrease apoptosis (Khan et al., 2019). ATM and ATR are sensors of DNA lesions that recognize failed DNA repairs and excessive DNA lesions and consequently trigger apoptosis (Roos and Kaina, 2013). In addition, YAP1 mediates mitochondrial homeostasis to resist ischemic stress. YAP1 can activate optic atrophy 1 (OPA-1)-mediated mitochondrial fusion, which ensures mitochondrial homeostasis (Ma and Dong, 2019). Mitochondrial fusion can reduce IR-induced mitochondrial fragmentation and mitochondrial oxidative stress, and suppress mitochondrial apoptosis; this promotes cell survival and alleviates cardiac IR injury (Anzell et al., 2018; Ma and Dong, 2019). *Park2* (a YAP1 target gene) encodes Parkin, which is an important E3 ubiquitin ligase in the outer mitochondrial membrane. Parkin promotes the clearance of impaired mitochondria through mitochondrial autophagy, which enhances stress resistance of CMs (Kubli et al., 2013; Leach et al., 2017). Moreover, YAP1 binding to Forkhead box protein O1 (FoxO1) was shown to facilitate antioxidant catalase and manganese superoxide dismutase transcription; the consequent decrease in ROS generation was shown to protect CMs against IR-induced oxidative stress (Shao et al., 2014). In the setting of MI, heterozygous inactivation of YAP1 in heart enhances CM apoptosis, fibrosis, enlarges infarct size, and impairs cardiac function; YAP1 activation using adeno-associated virus subtype 9: human YAP1 (AAV9: hYAP1) can alleviate MI injury and ameliorate cardiac function (Lin et al., 2014). In contrast to IR, YAP1 exerts anti-apoptosis activity via activation of PI3K-AKT signaling and increases CM proliferation against MI damage. Therefore, YAP1 mainly suppresses cell apoptosis through several downstream pathways and outputs to protect CMs and the heart from IR and MI damage.

### Role of Hippo–YAP1/TAZ Signaling in Cardiac Pressure Overload

Similar to IR damage, endogenous RASSF1A phosphorylates and activates MST1 to promote CM apoptosis and impair cardiac function following transverse aortic constriction (TAC) - a form of PO (Del Re et al., 2010). This RASSF1A-induced apoptosis can be inhibited by MST1 deficiency. In the heart with CM-specific KO of RASSF1A, endogenous MST1 activation is abolished, and there is reduced apoptosis, fibrosis, and hypertrophy after TAC (Del Re et al., 2010). Thus, the RASSF1A-MST1 pathway is implicated in causing heart damage in response to PO stress. Another component of the Hippo pathway, LATS2, is elevated and activated after TAC. Cardiac overexpression of LATS2 was shown to be associated with diminished left ventricular systolic and diastolic dysfunction and smaller left and right ventricles (Matsui et al., 2008). Overexpression of dominant negative LATS2 (DN-LATS2) in the murine heart was found to repress CM apoptosis and promote hypertrophy following TAC (Matsui et al., 2008). In addition, a recent study showed activation of endogenous YAP1 (the terminal effector of the Hippo pathway) in the compensated phase in response to TAC (Byun et al., 2019). Heterozygous cardiac-specific YAP1 KO mice, when subjected to TAC, present blunted cardiac hypertrophy and amplified CM apoptosis and fibrosis, with resultant cardiac dilatation and dysfunction (Byun et al., 2019). The Hippo pathway, as a deleterious regulator, has been shown to exacerbate PO-induced cardiac damage; therefore, inhibition of the Hippo pathway may have a cardioprotective effect against PO injury. Mechanistically, YAP1 directly triggers the transcription of target genes to mediate downstream output and to protect the heart against PO damage. *miR-206* is identified as the novel target gene of YAP1; thus, YAP1-induced miR-206 expression promotes cell hypertrophy and cell survival by downregulating the expression of the Forkhead box protein P1 (FoxP1) (Yang et al., 2015). YAP1, through its regulation of miR-206, promotes cell hypertrophy and exerts a cardioprotective effect in response to TAC. Moreover, YAP1 promotes *Pik3cb* transcription to activate PI3K-AKT signaling and trigger CM proliferation and survival (Xin et al., 2011; Lin et al., 2015). Collectively, these studies demonstrate the cardioprotective role of YAP1/TAZ against several stressors. Thus, YAP1/TAZ may serve as a therapeutic target in many cardiac diseases.

### Pernicious Role of YAP1/TAZ in Cardiac Disease

Although YAP/TAZ undertake the cardioprotective role against cardiac damage, high levels of YAP1/TAZ activity are not always beneficial. The effects of long-term suppression of Hippo signaling or activation of YAP1/TAZ in cardiac disease are opposite to those of short-term suppression. Clinical ischemic heart disease and idiopathic dilated cardiomyopathy (DCM) is characterized by enhanced YAP1/TAZ protein levels and increased transcriptional activity with the resultant upregulation of target genes, such as *ankyrin repeat domain 1*, *CTGF*, and *CYR61*; moreover, this phenomenon was observed in the murine heart with desmin-related cardiomyopathy (Hou et al., 2017). The WW45 CKO by Myh6-Cre mice show sustained YAP1 activation in CMs, which induces cardiac dysfunction, severe heart failure, and enhanced mortality in response to TAC (Ikeda et al., 2019a, b). CMs are subject to cell-cycle re-entry, increased de-differentiation, and decreased apoptosis; however, heart with active YAP1 cannot be rescued from PO damage (Ikeda et al., 2019a, b). This may be explained by the dual role of oncostatin M (OSM), which is identified as a novel target of the YAP1-TEAD1 complex. OSM promotes CM de-differentiation and induces the expression of stem cell markers to improve CM resistance to damage; however, excessive OSM stimulation inhibits CM contractility and induces cardiac remodeling (Kubin et al., 2011). In addition, YAP1-dependent OSM expression further activates YAP1 by inhibiting LATS2-induced phosphorylation, thereby forming a positive feedback loop (Ikeda et al., 2019a). This positive feedback mechanism results in sustained YAP1 activation and redundant OSM, which exacerbates cardiac injury and heart failure upon PO. Furthermore, the YAP1/TEAD1-OSM feedback cycle develops in the heart of high-fat-diet (HFD)-fed mice following PO, and contributes to the progression of diabetic cardiomyopathy (Ikeda et al., 2019b).

These observations suggest a pleiotropic role of YAP1 (Ikeda et al., 2019b). YAP1 activation during the acute phase of CM injury can induce compensatory cell proliferation and de-differentiation, which temporarily improves the tolerance of CM to damage. In later periods, persistent activation of YAP1 produces cardiac dysfunction and heart failure, which may be partly caused by the YAP1/TEAD1–OSM positive feedback loop and cardiac remodeling. However, these events do not completely explain the cardiac pathology, and there is a need to further explore the underlying mechanisms. The available evidence suggests the significance of sustaining the appropriate YAP1/TAZ protein level in therapy of cardiac disease.

The involvement of the Hippo pathway in cardiac fibrosis and remodeling explains the pernicious role of YAP1/TAZ in cardiac disease. In a murine DCM model, endogenous Angiotensin II (Ang II) was shown to activate YAP1 and promote the proliferation of cardiac fibroblasts and their transdifferentiation to myofibroblasts; subsequently, these changes were found to induce cardiac remodeling and impaired cardiac contractility (Jin et al., 2019). The Hippo–YAP1/TAZ pathway, as a mechano-sensor, is affected by mechanical signaling through cell geometry and alterations in cytoskeletal tension; this may help elucidate the pathological mechanism of chronic heart failure (Dupont et al., 2011). The subcellular localization and activity of YAP1/TAZ varies in cells with different matrix stiffness. In a stiff matrix, there is nuclear translocation and higher transcriptional activity of YAP1/TAZ; in contrast, a soft matrix is characterized by cytoplasmic sequestration and reduced activity of YAP1/TAZ (Nasrollahi and Pathak, 2017). The above studies potentially support the hypothesis that, in the context of cardiac ECM remodeling and/or fibrosis, the ECM stiffness and/or cytoskeletal tension may change and affect YAP1 activity; furthermore, activated YAP1 modulates the progression of ECM remodeling and fibrosis, which further exacerbates cardiac injury and even contributes to the development of chronic heart failure. However, the relationship between mechanical stimuli, remodeling/fibrosis, and YAP1/TAZ is not well characterized; therefore, studies to identify YAP1/TAZ activity and effects in cardiac disease are urgently needed.

## Role of Hippo–YAP1/TAZ Signaling in Cardiac Regeneration

Several endogenous and exogenous factors may cause cardiac damage and induce cardiac dysfunction. The heart is a highly differentiated organ with limited regenerative activity; this makes it difficult to restore normal function after any injury (Moya and Halder, 2018). Moreover, the regenerative capability of the adult heart is less than that of the neonatal heart. Consequently, cardiac regeneration can be activated through re-entry of terminally differentiated cells into cell cycle, reactivation of cell proliferation, and/or differentiation of stem cells into CMs, which contributes to post-damage recovery (Tables 2, 3; Ponnusamy et al., 2017; Moya and Halder, 2018).

As a pathway that controls organ size, Hippo–YAP1/TAZ signaling is essential for cardiac regeneration and could be applied for cardiac regeneration and targeted therapeutics in cardiac disease. The downstream effectors of YAP1/TAZ have clear biological effects in cardiac regeneration and CM protection. Studies have shown that adult and postnatal hearts with the deletion of SAV or LATS1/2 exhibit renewal capacity, which contributes to recovery after MI and cardiac apex resection (Heallen et al., 2013; Leach et al., 2017). Postnatal hearts lacking Hippo exhibit increase in cell number and myocardial regeneration following cardiac apex resection; post-MI, SAV CKO hearts show increased LV CMs, less fibrosis, and improved cardiac function (Heallen et al., 2013; Leach et al., 2017). Mechanically, SAV deletion was found to upregulate cell cycle genes and heart growth-related genes, which increases DNA synthesis and induces cell-cycle re-entry (Leach et al., 2017). Among these upregulated genes, *Park2* (encoding Parkin) was found to promote cell regeneration following stress. Parkin participates in the clearance of damaged mitochondria through mitochondria autophagy and apoptosis, and decreases the CM sensitivity to ischemic stress (Kubli et al., 2013; Leach et al., 2017). Additionally, studies have demonstrated enhanced expression of Paired-like homeodomain transcription factor 2 (Pitx2) in the Hippo-deficient heart (Tao et al., 2016). Pitx2, as a transcriptional factor, has the capacity to regulate several target genes encoding mitochondrial, oxidation-reduction, and respiratory chain proteins via binding to YAP1 or other undefined co-factors. Therefore, Pitx2 and YAP1 may synergistically trigger anti-oxidative effect and proliferation activity to improve the stress resistance and regeneration of adult and neonatal heart (Shao et al., 2014; Tao et al., 2016). These findings suggest that the Hippo pathway plays the role of a pernicious mediator in the context of cardiac regeneration.

The YAP1 CKO neonatal heart lacks healthy myocardial tissue in the LV wall and shows enhanced fibrotic infarct zone following an MI; neonatal heart expressing active form of YAP1 exhibits increased myocardial tissue and reduced LV fibrosis (Xin et al., 2013a). Thus, the proliferative activity of YAP1 is essential for cardiac regeneration, and its regenerative activity is involved in proliferative gene programs. Mechanically, glycolytic YAP1 activation upregulates miR-152 expression to increase CM proliferation in neonatal mice (Wang et al., 2018). Furthermore, miR-152 suppresses the expression of cell-cycle inhibitory proteins, such as p27kip and DNA methyltransferase1 (DNMT1), leading to re-entry of neonatal CMs in the cell cycle. YAP1 activation promotes cell proliferation not only directly through targeting of the *CTGF* but also indirectly through inhibition of cell-cycle-inhibitory proteins; this contributes to neonatal cardiac regeneration and repair after MI injury (Haskins et al., 2014; Wang et al., 2018). As mentioned in Section “Role of Hippo–YAP1/TAZ Signaling and Its Differentiation Output in Heart Development,” YAP1/TAZ have the capacity to induce proliferation and de-differentiation through targeting of the related genes (*Pik3cb*, *Dpp4*, *Dhrs3*, *SOX17*, *Bmp2b*, and others), which may induce CMs to re-enter the cell cycle and regeneration (Billings et al., 2013; Khurana et al., 2013; Lin et al., 2015; Estaras et al., 2017; Fukui et al., 2018; Hsu et al., 2018; Xiao et al., 2018).

In addition to their action on highly differentiated CMs, YAP1/TAZ regulate stem cells to undergo differentiation and proliferation to achieve cardiac regeneration. P2Y_2_ receptor (P2Y_2_R) is a pro-regenerative GPCR that regulates cell regeneration after damage (Vassort, 2001). Activated P2Y_2_R promotes the proliferation and migration of human c-Kit^+^ cardiac progenitor cells (hCPCs) via activation of YAP1 (Khalafalla et al., 2017). Moreover, hCPCs improves cardiac function in MI animal models, which supports the use of hCPCs for stem cell therapy in cardiac failure (Ellison et al., 2013). YAP5SA, an active version of YAP1, is overexpressed in the mouse heart. YAP5SA overexpression causes CMs to partially re-enter the cell cycle; in addition, CMs can be reprogrammed into a more primitive and fetal cell state, whereby the heart exhibits thickened ventricular walls and smaller chambers (Monroe et al., 2019). Active Yes phosphorylates YAP1 on one or more tyrosine residues; subsequently, phosphorylated YAP1 translocates into the nucleus and induces the transcription of the key pluripotency factor *Oct-3/4* (Tamm et al., 2011). Oct-3/4 is critical for maintaining the self-renewal capacity of mouse and human ESCs. It promotes the cell cycle re-entry of ESCs via suppressing the transcription of cyclin-dependent kinase inhibitors (Lee et al., 2010). These findings indicate that YAP1 plays a critical role in maintaining the proliferative and de-differentiation capacity of stem cells and promoting adult CM renewal following injury.

Yes-associated protein 1/TAZ have the capacity to reactivate CM proliferation and may play a role in cardiac regeneration and therapy for cardiac disease. Nonetheless, the complete mechanism of action and the effects of YAP1/TAZ in cardiac development and disease remain an enormous puzzle. Therefore, further research is required to unravel the contributory mechanisms in order to clearly understand the therapeutic potential of targeted activation of the YAP1/TAZ signaling pathway in myocardial disease and cardiac regeneration.

## Compounds That Regulate Hippo–YAP1/TAZ Signaling

Several reports have focused on the role of Hippo–YAP1/TAZ signaling in cardiac disease to explore the efficacy of leading therapeutic compounds and agents. The YAP1/TAZ–TEAD complex is considered a novel drug target to affect its biological output. Verteporfin, an inhibitor of YAP1, has been widely used to study the Hippo–YAP1/TAZ pathway (Khalafalla et al., 2017; Ikeda et al., 2019b). Verteporfin combined with YAP1 prevents the interaction of the YAP1-TEADs complex; verteporfin alters the conformation of YAP1 to enhance its binding with trypsin as well as tryptic cleavage (Liu-Chittenden et al., 2012; Tang et al., 2019). As mentioned in Section “Role of Hippo–YAP1/TAZ Signaling and Its Differentiation Output in Heart Development,” YAP1/TEAD1-OSM feedback cycle exacerbates heart failure and the progression of diabetic cardiomyopathy in HFD-fed mice following TAC (Ikeda et al., 2019b). Verteporfin treatment via suppressing YAP1/TEAD1 complex alleviates cardiac dysfunction and improves the survival rate of HFD-fed mice by increasing the expression of CM dedifferentiation protein and by attenuating myocardial infiltration. P2Y_2_R-induced YAP1 activation promotes the proliferation and migration of hCPCs, which can be repressed by verteporfin (Khalafalla et al., 2017). Therefore, verteporfin may abolish the capacity of hCPCs in repairing MI injury (Ellison et al., 2013). YAP1/TAZ luciferase reporter assays have identified apigenin as a potential YAP1 inhibitor; it was found to disrupt the interaction between YAP1 and TEAD (Li et al., 2018b). Apigenin, a kind of flavonoid, was found to exhibit a protective effect against cardiovascular diseases. *In vivo* and *vitro*, apigenin suppresses ROS production, loss of mitochondrial membrane potential (MMP), and apoptosis via PI3K/AKT signaling as well as mitochondrial Notch1/Hes1 signaling; this was found to protect H9C2 cells and rat hearts against IR injury (Hu et al., 2015; Zhou et al., 2018). However, there is no evidence to suggest that apigenin can mediate the Hippo–YAP1/TAZ pathway to defend heart against stress.

Yes-associated protein 1/TAZ are affected by other pathways; in addition, certain drugs have been shown to target the upstream regulation of YAP1. Dasatinib, which targets the mitogen-activated protein kinase (MAPK) pathway (a pro-proliferative pathway, in contrast to Hippo signaling), may affect YAP1 phosphorylation and inhibit its activity (Rosenbluh et al., 2012; He et al., 2016). Inhibition of YAP1 by dasatinib was found to induce differentiation of hESCs into APS-derived endoderm and cardiac mesoderm, consequently affecting the progression of cell differentiation in cardiogenesis (Hsu et al., 2018). GPCRs are targeted by extracellular ligands and regulate YAP1 activity. As mentioned in Section “Role of Hippo–YAP1/TAZ Signaling and Its Differentiation Output in Heart Development,” Ang II and melatonin activate YAP1 through GPCRs to induce corresponding changes in cardiac function (Jin et al., 2019; Ma and Dong, 2019). YAP1 is activated by endogenous Ang II to promote the proliferation of cardiac fibroblasts and their transdifferentiation to myofibroblasts, inducing cardiac remodeling and DCM (Jin et al., 2019). In addition, melatonin facilitates OPA-1-mediated mitochondrial fusion via activating YAP1, which attenuates IR-induced mitochondrial apoptosis and alleviates cardiac IR damage (Ma and Dong, 2019). Dobutamine enhances YAP1 Ser127 phosphorylation and cytoplasmic sequestration through β-adrenergic receptor (a class of GPCRs) rather than the Hippo pathway (Bao et al., 2011). A35 (an antitumor compound) induces mutation of YAP1 Ser127, which induces the recovery of proliferative inhibition and apoptosis compared to wild-type YAP1 (Zhao et al., 2018). Although Dobutamine and A35 can mediate YAP activity in cancer cells, their effects on YAP1 in CMs and heart tissue are yet to be elucidated.

In conclusion, drugs and compounds that control kinases upstream and/or downstream of YAP1/TAZ can regulate YAP1/TAZ activity and subsequently influence cardiac injury. Undoubtedly, YAP1/TAZ is a potential therapeutic target for cancer and cardiac disease. However, the latent regulatory network of YAP1/TAZ has not been completely elucidated in previous research, especially in the context of cardiac disease.

## Author Contributions

NH, XC, and YL participated in research design. NH and JL supervised in research design. XC and YL performed the data analysis. NH, JL, XC, and YL wrote or contributed to the writing of the manuscript.

## Conflict of Interest

The authors declare that the research was conducted in the absence of any commercial or financial relationships that could be construed as a potential conflict of interest.
